# Assessing Patient Awareness and Misconceptions Regarding MRI Radiation Risks in China: A Questionnaire Study

**DOI:** 10.1002/nop2.70657

**Published:** 2026-07-15

**Authors:** Jiaojiao Chen, Siyuan Yang, Jianying Li, Jiahui Wu, Mingzhao Xiao, Li Tong

**Affiliations:** ^1^ Department of Radiology The First Affiliated Hospital of Chongqing Medical University Chongqing China; ^2^ Center of Nursing Research The First Affiliated Hospital of Chongqing Medical University Chongqing China

**Keywords:** contrast agent awareness, healthcare communication, MRI safety, patient education, radiation misconceptions

## Abstract

**Aim:**

This study aimed to assess Chinese patients' perceptions regarding the safety of magnetic resonance imaging (MRI) and its contrast agents, with a particular focus on their understanding of radiation exposure. While misconceptions regarding the safety of MRI examinations are well documented among Western patient populations, relevant evidence from China remains limited.

**Design:**

A descriptive cross‐sectional study.

**Methods:**

A cross‐sectional survey was administered to 272 patients at a tertiary hospital in China. Data were collected using a self‐administered questionnaire derived from MRI safety literature. The questionnaire comprised four sections: demographic information (Part A), MRI radiation safety perceptions (Part B), contrast agent safety knowledge (Part C), and preferred information sources (Part D).

**Results:**

272 patients completed the questionnaire, with common misconceptions regarding MRI‐related safety knowledge observed in this study. 60.7% patients erroneously believed MRI utilizes ionizing radiation, and only 15.4% correctly recognized the non‐radioactive nature of contrast agents. While gender showed no significant association with fundamental MRI safety knowledge (*p* > 0.05), female participants demonstrated significantly greater accuracy regarding contrast agent safety (*p* = 0.0048). Notably, healthcare providers constituted the preferred information source for 68.01% of respondents.

**Conclusion:**

Chinese patients exhibit prevalent safety misconceptions concerning MRI radiation properties and contrast agent mechanisms, and they most expect to receive health education from medical professionals. Standardized, multi‐level collaborative health education interventions may help rectify such knowledge gaps, which could potentially lead to better patient adherence and imaging efficiency.

**Implications for Nursing Practice:**

Nurses may consider implementing standardized health education programs to address patients' misunderstandings about MRI safety and contrast agent radiation risks.

**Patient or Public Contribution:**

No patient or public contribution.

## 1 Introduction

Magnetic Resonance Imaging (MRI) has become a cornerstone of modern diagnostic medicine due to its non‐ionizing radiation and high‐resolution soft tissue imaging. When combined with intravenous contrast agents, MRI significantly improves the ability to distinguish normal from diseased tissues, thereby enhancing diagnostic accuracy (Scarciglia et al. 2025; Chandra and Mohan 2016; Dewey and Kitterick 2021). Its global popularity attests to its practicality, with 34 million MRI scans performed annually in the United States alone (Gajawelli et al. 2019).

However, patients' insufficient understanding of the safety of MRI constitutes a key obstacle to optimizing its clinical application. Multiple studies have revealed widespread misconceptions. For example, a survey in Ghana showed that 52% of participants mistakenly believed that MRI exposure could cause cancer (Asante and Acheampong 2021). These misunderstandings not only exacerbate patients' anxiety but may also lead to scan cancellations or image artefacts due to patient motion (Darren et al. 2025; Ajam Amna et al. 2020).

The complexity of MRI safety issues stems from multiple factors, including the use of contrast agents, the potential risks associated with strong electromagnetic fields, and individual patient factors such as implanted devices (Alahmari et al. 2022; Watson Robert and Yu 2023). Notably, patients often mistakenly attribute radiation risks to MRI, confusing it with other ionizing radiation technologies such as CT scans. This phenomenon has been observed in studies conducted in Turkey and the UK (Duymuş et al. 2016; Kumar and Irving 2020). Guidelines from Canada and other countries emphasize that evidence‐based patient education is crucial for correcting these misconceptions, particularly for vulnerable groups such as pregnant women (Barrett et al. 2024).

The existing literature on patients' perceptions of MRI predominantly originates from regions such as Africa (Darren et al. 2025), Europe (Watson Robert and Yu 2023) and North America (Duymuş et al. 2016), with a primary focus on non‐contrast‐enhanced MRI applications. In contrast, research specifically addressing contrast‐enhanced MRI (CE‐MRI) and the safety of contrast agents remains markedly scarce. Within China, this field has yet to be sufficiently explored. Given China's large population base, rapidly expanding healthcare system, and marked regional disparities in cultural, educational, and economic factors, patients' perceptions of MRI safety particularly regarding CE‐MRI may exhibit distinct characteristics. Should misconceptions regarding MRI safety exist within the Chinese patient population, the implementation of targeted educational interventions may potentially contribute to better patient compliance and procedural safety. Therefore, this study was designed to preliminarily investigate Chinese patients' pre‐scan perceptions of the MRI examination process and contrast agent safety. The findings may serve as an evidence base for the development of tailored patient education strategies, which may potentially support patient compliance and inform MRI clinical practice within the Chinese healthcare system.

## 2 Patients and Methods

This descriptive cross‐sectional study was carried out at a tertiary hospital in China. The study was conducted in accordance with the Declaration of Helsinki and was approved by the Ethics Committee of The First Affiliated Hospital of Chongqing Medical University (approval no. 2022‐414‐01, approved on 20 November 2022). 272 participants were chosen using convenience sampling from May to June 2023. Conscious adult patients receiving CE‐MRI were included, and emergency, coma, sedation, and other uncooperative patients were excluded. Signed informed consent was obtained from all participants. Participants were asked to complete a self‐administered questionnaire designed after reviewing related studies. To minimize recall bias and reporting bias, the questionnaire was designed with clear and neutral items, and participants were informed that all responses were anonymous and that there were no right or wrong answers.

The questionnaire had four parts. Part A aimed to gather the basic patient information such as gender, age, occupation, marital status, etc. Part B designed to assess the patients' perceptions of safety in MRI examination, comprising five single‐choice questions with response options of “yes”, “no”, or “unclear”. One correct answer was awarded one point, and the rest were not awarded, with a total of 5 points. Part C targeted to evaluate the patient's perception of the contrast agent used in MRI. This section consisted of four single‐choice questions, with each offering three response options: “yes”, “no”, and “unclear”. One correct answer gets one point, and the rest gets no extra points, for a total of 4 points. Part D comprised ways in which patients expect to acquire knowledge about examinations, including medical staff, social media, lectures, manuals, and newspapers, etc. The participants completed a hard‐copy questionnaire only once. Deleted invalid questionnaire before data analysis, including incomplete answers, duplicate responses across items and dummy entries.

### 2.1 Data Analyses

Descriptive statistics were calculated using frequency, percentage, mean, and standard deviation. Chi‐square tests were used to assess gender differences in the distribution of categorical variables. Due to the non‐normal distribution of questionnaire B and C scores, the Wilcoxon rank‐sum test was used to evaluate the effect of gender on these scores. *p* < 0.05 was considered significant. All statistical analyses were performed using SAS 9.4 (SAS Institute Inc., Cary, NC, USA).

## 3 Results

A total of 272 valid questionnaires were included in the final analysis, consisting of 177 female and 95 male participants. Baseline demographic characteristics were summarized in Table 1. Overall, most participants were middle‐aged, predominantly of Han ethnicity, resided in urban areas, and were married, with a considerable proportion having a high school or technical secondary school educational background. Approximately 20% of patients had previously undergone CE‐MRI examination three times or more. On the variables listed, the difference between men and women was not statistically significant.

**TABLE 1:** Personal data of survey respondents.

Variables	No (%)			*p*
	Women (*N* = 177)	Men (*N* = 95)	Total (*N* = 272)	
Age				0.0585
18–40	54 (30.51)	24 (25.26)	78 (28.68)	
41–60	107 (60.45)	53 (55.79)	160 (58.82)	
> 60	16 (9.04)	18 (18.95)	34 (12.50)	
Education				0.5895
Junior high school and below	66 (37.29)	30 (31.58)	96 (35.29)	
High school/technical secondary school	41 (23.16)	22 (23.16)	63 (23.16)	
College or above	70 (39.55)	43 (45.26)	113 (41.54)	
Marital status				0.4096
Married	148 (83.62)	83 (87.37)	231 (84.93)	
Other	29 (16.38)	12 (12.63)	41 (15.07)	
Ethnic				0.2382
Han	167 (94.35)	86 (90.53)	253 (93.01)	
Other	10 (5.65)	9 (9.47)	19 (6.99)	
Residence				0.5786
Urban	125 (70.62)	64 (67.37)	189 (69.49)	
Rural	52 (29.38)	31 (32.63)	83 (30.51)	
Previous CE‐MRI examination times				0.6275
1–2 times	141 (79.66)	78 (82.11)	219 (80.51)	
≥ 3 times	36 (20.34)	17 (17.89)	53 (19.49)	

The survey results indicated that a significant number of patients held misconceptions regarding the safety of MRI and contrast agents. More than 60% of the respondents erroneously believed that MRI involved radiation and posed health risks, while only about 13% provided the correct answer and 20% reported being uncertain. Additionally, approximately 30% of patients thought that a single MRI scan produced higher ionizing radiation than a CT or plain X‐rays, and that safety precautions were required before an MRI scan. Similarly, only around 15% of respondents correctly answered that the contrast medium was non‐radioactive and harmless to health, while over 55% of patients indicated they were unsure. About 30% of patients incorrectly believed that contact with children or pregnant women should be avoided after contrast agent administration.

Table 2 showed the overall scores of part B and C. The MRI safety cognition score of female patients was 2.10 ± 1.48, while that of male patients was 2.02 ± 1.29. The perception of MRI safety was similar between men and women, with no significant gender difference (*p >* 0.05). The contrast agent safety cognition scores of female patients and male patients were 2.27 ± 1.04 points and 1.72 ± 0.88 points respectively. Further analysis showed that the *p*‐value was 0.0048, less than 0.05, indicating that women had a higher perception of the safety of contrast agents than men, and the difference was statistically significant.

**TABLE 2:** Patient safety perception of MRI and contrast agent.

Variables	Mean (std)			*Z* ^a^	*p*
	Women (*N* = 177)	Men (*N* = 95)	Total (*N* = 272)		
MRI	2.10 (1.48)	2.02 (1.29)	2.08 (1.42)	0.0919	0.9268
Contrast agent	2.27 (1.04)	1.72 (0.88)	2.12 (1.02)	−2.8232	0.0048

^a^ Wilcoxon rank sum test.

According to the survey on patients' preferred MRI safety information sources (Table 3), medical professionals, the internet or social media, and booklets ranked as the top three. Further analysis indicated that female patients showed a significantly higher preference for lectures or training than male patients, while no significant gender differences were observed in other information acquisition preferences.

**TABLE 3:** Prefer access to receive information.

Variables	No (%)			*p*
	Women (*N* = 177)	Men (*N* = 95)	Total (*N* = 272)	
Health care staff				0.8670
Yes	121 (68.36)	64 (67.37)	185 (68.01)	
No	56 (31.64)	31 (32.63)	87 (31.99)	
Internet or social media				0.0671
Yes	97 (54.80)	41 (43.16)	138 (50.74)	
No	80 (45.20)	54 (56.84)	134 (49.26)	
Lecture/Training				0.0417
Yes	58 (32.77)	20 (21.05)	78 (28.68)	
No	119 (67.23)	75 (78.95)	194 (71.32)	
Booklets				0.4558
Yes	81 (45.76)	39 (41.05)	120 (44.12)	
No	96 (54.24)	56 (58.95)	152 (55.88)	
Television/radio				0.4833
Yes	40 (22.60)	18 (18.95)	58 (21.32)	
No	137 (77.40)	77 (81.05)	214 (78.68)	
Newspapers				0.0685
Yes	36 (20.34)	11 (11.58)	47 (17.28)	
No	141 (79.66)	84 (88.42)	225 (82.72)	
Communication with patients				0.6367
Yes	30 (16.95)	14 (14.74)	44 (16.18)	
No	147 (83.05)	81 (85.26)	228 (83.82)	

## 4 Discussion

Currently, there is relatively limited literature on the MRI safety awareness among Chinese patients. As an exploratory study, this research preliminarily addressed this under‐investigated field. This study's key finding revealed that the enrolled patients exhibited prevalent misconceptions regarding the safety of MRI and contrast agents. There was no significant difference between men and women in the basic MRI safety cognition (radiation, harm), but in the contrast agent safety cognition, female patients scored significantly higher than men. In terms of patient information acquisition preferences, medical staff were the primary source for participants, while digital media and manuals showed promising application potential.

This survey indicated that participants in this study exhibited widespread misconceptions regarding MRI safety. Over 60% of participants misperceived MRI as a source of ionizing radiation, a finding consistent with prior studies demonstrating similar misinterpretation rates (77% in Ghanaian patients (Dewey and Kitterick 2021); 62% in Turkish cohorts (Duymuş et al. 2016)). This confusion may partly stem from the conflation of MRI with other conventional imaging examinations like CT and plain X‐rays, which do involve ionizing radiation. Imaging examinations may be frequently misassociated with radiation exposure in public perception. The situation may be exacerbated by the Chinese term for MRI, “hé cí gòng zhèn”, as the character referring to “nuclear” may prompt false assumptions of radiation exposure among patients. This misunderstanding is reflected in the findings that approximately 30% of participants incorrectly believed that a single MRI examination delivers higher radiation exposure than CT or plain X‐rays, which is broadly consistent with the results of earlier studies (Duymuş et al. 2016; Ria et al. 2017). As a result, patients may perceive unnecessary safety precautions as essential, which may appear inconsistent with established scientific evidence. Such misconceptions may potentially contribute to elevated anxiety, avoidance of clinically indicated MRI examinations, and demands for unnecessary protective precautions. In turn, these issues might potentially affect patient cooperation and could contribute to greater complexity in clinical imaging procedures.

This study revealed common patient misunderstandings about the safety of contrast agents. Among the enrolled participants, only 15.4% correctly recognized that contrast agents are non‐radioactive, whereas 60.3% reported uncertainty and 24.3% held the false belief that these contrast agents emit radiation. Furthermore, about 30% of the patients thought that close contact with children or pregnant individuals should be avoided after contrast agent injection, a trend that is in line with the findings of a previous study (Shrestha and Khadka 2020). This may suggest a common misconception among patients linking injectable agents to radioactivity, as individuals tend to confuse MRI contrast agents with radioactive tracers applied in nuclear medicine examinations. Such misunderstandings may be associated with patients' reluctance to receive contrast‐enhanced procedures and may add to their psychological burden and possibly extend scanning duration (Alahmari et al. 2022; Shrestha and Khadka 2020). The actual potential risks primarily include rare allergic reactions and nephrogenic systemic fibrosis, which dominantly affect high‐risk patients with renal impairment (Chandra and Mohan 2016). The implementation of multi‐level health education may help alleviate such cognitive biases. The formulation of a unified MRI safety education guideline could standardize education content across medical institutions at all levels and promote its standardization. Digital platforms such as WeChat and short video applications may support the targeted delivery of professional knowledge related to imaging examinations, which could reduce public cognitive misconceptions. Furthermore, strengthened collaboration among medical institutions, health authorities, and communities to carry out regular public science popularization activities may contribute to improving the general public's overall literacy of imaging safety cognition. Notably, although female patients exhibited significantly higher knowledge scores than male counterparts (2.27 ± 1.04 vs. 1.72 ± 0.88; *p* = 0.0048), which aligns with cross‐cultural trends of women's active engagement in health information acquisition, this gender‐based difference appeared insufficient to alleviate the core misconceptions identified in this study (Alelyani et al. 2021).

Although patients regard healthcare providers as their primary source of medical imaging knowledge, there may be discrepancies between their perceived reliance on information sources and their actual information acquisition behaviours. Studies have shown that most patients obtain relevant information from family and friends rather than from their health care providers, and have never discussed radiation risks with them. This may be due to factors such as limited doctor‐patient communication time and the use of complex medical terminology (Asante and Acheampong 2021; Ria et al. 2017; Lumbreras et al. 2016). Therefore, optimizing doctor‐patient communication in the radiology department and across the whole hospital may be valuable. Integrating MRI safety education into routine clinical procedures could enable regular professional publicity during examination ordering and examination preparation. Specialized training for medical staff may help consolidate their professional knowledge related to imaging examinations and MRI safety. Leveraging the professional authority of medical workers, hospital‐based face‐to‐face education, supplemented by diversified intelligent education modes, can help address patients' concerns and reduce related misunderstandings.

Among Supporting Information channels, the internet and social media exhibit relatively high utilization yet contain substantial misleading information, which is consistent with the findings of the earlier study (Ria et al. 2017). Healthcare institutions and professional societies may consider proactively disseminating authoritative, accessible educational materials through official websites, short video platforms, and other relevant channels. Printed brochures remain a viable supplementary educational tool. Integrating core imaging knowledge with visual design elements and strategically placing these materials in high‐traffic patient areas, including registration counters and waiting rooms, may help improve information accessibility. Although lectures and training programs show relatively low overall demand, they may offer considerable educational value for specific populations (e.g., female populations) and community health initiatives, facilitating in‐depth scientific health communication. In contrast, traditional media and patient peer‐exchange channels present limited utilization, indicating a reduced priority for targeted resource allocation. In summary, developing a multi‐modal information dissemination strategy focuses on two core objectives: improving the communication capabilities of healthcare providers (Shrestha and Khadka 2020) and establishing authoritative digital science communication platforms. Concurrent optimization of patient educational materials may help mitigate relevant misconceptions and could potentially enhance the accuracy and efficacy of health information delivery.

## 5 Conclusion

This study performed an exploratory assessment of Chinese patients' awareness of the safety of MR and contrast agents. The results indicated that enrolled patients generally have relevant cognitive misconceptions, and medical staff are the primary channel that patients prefer for obtaining related information. Enhancing the professional competency training of clinical staff, integrating standardized and personalized health guidance throughout the MRI examination process, and leveraging multi‐party collaboration to promote public education on imaging‐related knowledge could help correct patients' misconceptions and potentially support their awareness of MRI and contrast agent safety, which may contribute to better clinical compliance.

## 6 Limitations

This study has certain limitations. First, the reliability and validity of the survey tool need to be verified. Although the questionnaire was designed based on literature analysis and discussed by a team of radiology experts, it has not been systematically validated in the sample. Second, all data were collected via patient self‐report, which is susceptible to individual subjective perception and recall bias, inevitably causing certain reporting bias. Third, this single‐center study recruited participants from a single tertiary hospital in China using convenience sampling, without enrolling patients from other regions or primary medical institutions. The single‐source sample lacks sufficient representativeness and cannot fully reflect the actual cognitive levels of patients across different regions and healthcare institutions at various levels in China, which significantly limits the generalizability and extrapolability of the research findings. Thus, caution is warranted when applying or promoting the study findings. Future research will first conduct pilot testing of the questionnaire and assess its reliability and validity. A multi‐center, large‐sample study involving patient populations from hospitals of different tiers is needed to enhance the reliability and generalizability of the findings.

## 7 Implications for Nursing Practice

Nurses are encouraged to implement standardized health education programs to help alleviate patients' misunderstandings about MRI safety and the radiation risks of contrast agents. Relevant educational interventions can be integrated into key clinical procedures, such as appointment scheduling and contrast agent preparation, and delivered by trained nurses. Improved patient safety awareness and examination compliance may be associated with reduced motion artefacts, potentially better scanning efficiency, and more effective utilization of medical resources.

## Author Contributions

The authors confirm that they made substantial contributions to the conceptualization and design of the study, participated in data collection, and contributed to data analysis and interpretation. They also drafted and critically revised the manuscript for intellectual content, agreed to submit it to this journal, approved the final version for publication, and assumed full accountability for all aspects of the study.

## Funding

The authors have nothing to report.

## Disclosure

Artificial Intelligence (AI) Use Statement: The authors have nothing to report.

## Ethics Statement

This study was reviewed and approved by the Ethics Committee of The First Affiliated Hospital of Chongqing Medical University (approval no. 2022‐414‐01, approved on 20 November 2022). Data were anonymized for analysis, and the study was conducted in accordance with the principles of the Declaration of Helsinki.

## Consent

All participants provided written informed consent prior to the survey.

## Conflicts of Interest

The authors declare no conflicts of interest.

## Data Availability

The data that support the findings of this study are available from the corresponding author upon reasonable request.
